# The Evolution of Technology‐Driven In Vitro Models for Neurodegenerative Diseases

**DOI:** 10.1002/advs.202304989

**Published:** 2024-02-17

**Authors:** Eleonora De Vitis, Antonella Stanzione, Alessandro Romano, Angelo Quattrini, Giuseppe Gigli, Lorenzo Moroni, Francesca Gervaso, Alessandro Polini

**Affiliations:** ^1^ CNR NANOTEC—Institute of Nanotechnology Campus Ecotekn, via Monteroni Lecce 73100 Italy; ^2^ IRCCS San Raffaele Scientific Institute Division of Neuroscience Institute of Experimental Neurology Milan 20132 Italy; ^3^ Dipartimento di Medicina Sperimentale Università Del Salento Campus Ecotekne, via Monteroni Lecce 73100 Italy; ^4^ Complex Tissue Regeneration Maastricht University Universiteitssingel 40 Maastricht 6229 ER Netherlands

**Keywords:** bioinks, bioprinting, hydrogels, in vitro models, microfabrication, neurodegenerative diseases, organ‐on‐chip

## Abstract

The alteration in the neural circuits of both central and peripheral nervous systems is closely related to the onset of neurodegenerative disorders (NDDs). Despite significant research efforts, the knowledge regarding NDD pathological processes, and the development of efficacious drugs are still limited due to the inability to access and reproduce the components of the nervous system and its intricate microenvironment. 2D culture systems are too simplistic to accurately represent the more complex and dynamic situation of cells in vivo and have therefore been surpassed by 3D systems. However, both models suffer from various limitations that can be overcome by employing two innovative technologies: organ‐on‐chip and 3D printing. In this review, an overview of the advantages and shortcomings of both microfluidic platforms and extracellular matrix‐like biomaterials will be given. Then, the combination of microfluidics and hydrogels as a new synergistic approach to study neural disorders by analyzing the latest advances in 3D brain‐on‐chip for neurodegenerative research will be explored.

## Graphical abstract

Despite significant research efforts, the knowledge about neurodegenerative disorder pathological processes and the development of efficacious drugs are still limited due to the inability to access and reproduce the intricated microenvironment of the nervous system. The combined use of innovative technologies, such as organ‐on‐chip and 3D printing, represents a new synergistic approach to study neural disorders in vitro.

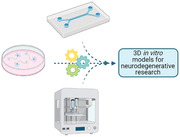

## 1 Introduction

Neurodegenerative diseases (NDDs) represent an umbrella term that includes more than 600 types of nervous system disorders, involving the central nervous system (CNS) as well as the peripheral nervous system (PNS). These conditions are defined by increasing loss of structure and function of neurons and, depending on the regions involved in this process, they can cause memory and cognitive dysfunction or motor impairment. Even though the onset of these disorders is thought to be associated with abnormalities at the molecular level, the exact causes are still unknown and most of them have not been studied yet due to the low frequency of cases in the world.^[^ ^1^ ^]^ The most studied among them are Alzheimer's disease (AD), Parkinson's disease (PD), and amyotrophic lateral sclerosis (ALS), which affect millions of people worldwide. It has been estimated that, in the past 15 years, the percentage of people affected by these diseases increased from 20% to 30%^[^ ^2^ ^]^ and, according to the World Health Organization, the incidence will keep rising, reaching more than double by 2050^[^ ^3^ ^]^ and likely becoming the second‐most prevalent cause of death. Their prevalence is increasing due to, in part, the extension of lifespan. At the moment, none of the NDDs is curable and the lack of effective treatments represents a critical public health and economic concern,^[^ ^4^ ^]^ since drugs available on the market attempt to ease symptoms and make life expectancy longer.^[^ ^5^ ^]^ As a consequence, the development of suitable models for the study of these disorders is necessary not only to better understand the biological pathways, but also to identify new potential drugs. Animal models for the study of brain development and neurodegenerative disorders were used as early as the 1950s and for years, especially humanized or transgenic mice, have been considered a gold standard for this purpose,^[^ ^6^ ^]^ but they cannot be generalized to humans. In fact, each species is unique in terms of lifespan, gene expression profiles,^[^ ^7^ ^]^ tissue‐specific cell turnover rates, and all these characteristics contribute to aging.^[^ ^6a^ ^]^ Furthermore, the anatomy and the physiology of the CNS and skeletal muscles (e.g., the spinal cord length) differ between species resulting in differences in disease phenotype and drug response.^[^ ^8^ ^]^ This discrepancy is the primary reason for the high failure rate of current drugs: in fact, it is estimated that this is up to 35% during phase I of clinical trials, 68% during phase II, and 30–40% at phase III. These percentages decrease further in the case of potential drug candidates for degenerative diseases, with only 15% of these molecules successfully completing phase I of clinical trials, and among them only 10% reaching the market.^[^ ^9^ ^]^ It is therefore clear that animal models for NDDs are not able to fully mimic human pathophysiology. Thus, to fill this gap it is essential to design innovative in vitro cell‐based models with a higher level of physiological relevance.

So far, both 2D/3D cell cultures and animal models^[^ ^10^ ^]^ have been employed in the field of NDDs, but all these approaches present various drawbacks. Conventional 2D cell cultures, such as Petri dishes and culture flasks, are widely used in the preclinical evaluation of drug candidates^[^ ^11^ ^]^ and in‐depth analysis of their biological effects due to the ease control and low‐cost maintenance of cell culture.^[^ ^12^ ^]^ However, their major limitation relies on the use of a single cell population in most cases, enriched with an additional cell type in a few cases.^[^ ^13^ ^]^ These systems are therefore rather primitive: cell–cell or cell–extracellular matrix (ECM) interactions, responsible for cell viability, differentiation, proliferation, and overall other aspects of cell behavior, are not represented. Moreover, in conventional neuronal cultures, neurons are randomly seeded, cultured in homogeneous media, and the separation between axons and somas fails. As a consequence, the data collected from 2D culture could provide deceptive information about pathological mechanisms and drug responses that are frequently over‐ or underestimated.^[^ ^14^ ^]^ On the other hand, 3D models overcome some of the limitations of 2D approaches since they reproduce in a more appropriate way the spatial and chemical complexity of living tissues. Traditionally, in fact, cells successfully grow in ECM analogues, such as hydrogels (i.e., matrigel, collagen, and fibrin), able to reproduce physiological microstructures. Numerous studies have shown that in 3D culture systems cells exhibit a behavior closer to the in vivo condition. This is because the 3D environment determines a spatial organization of the receptors on the cells by influencing and regulating signal transduction and gene expression.^[^ ^15^ ^]^ The interest in 3D culture systems is continuously growing and has shown great progress in the optimization of cell culture techniques,^[^ ^16^ ^]^ the synthesis, and characterization of new matrices to develop the 3D environment^[^ ^17^ ^]^ and the development of drug screening or discovery methods.^[^ ^18^ ^]^ Despite these benefits, 3D in vitro models suffer from some limitations: i) there is limited control on accurate cell positioning and, therefore, it is difficult to precisely study cell–cell communication;^[^ ^19^ ^]^ ii) it is quite difficult to compartmentalize neuronal networks or portions of the same cell (e.g., to separate neurites from their soma) in order to study degeneration processes strictly related to specific cell compartments; iii) cellular tension and mechanical stress (i.e., shear stress) cannot be fully reproduced;^[^ ^20^ ^]^ iv) sometimes they are not cost‐effective and protocols are not reproducible;^[^ ^21^ ^]^ and v) they are partially suitable for high‐resolution imaging and high‐throughput studies.^[^ ^22^ ^]^

Starting from the early 2010s, due to the advances in stem cell culture, spheroids, and organoids have become two innovative technological alternatives to the classic 3D models. Spheroids are multicellular aggregates that can generate spontaneously or be forced to form employing different techniques (e.g., hanging drop, magnetic levitation, and rotary cell culture). They show a specific geometry, with a dimension usually below 1 mm that allows oxygen and nutrient diffusion. However, they have limited self‐renewal and differentiation capability as they do not include stem or progenitor cells.^[^ ^23^ ^]^ An organoid can be considered as an advanced model of spheroid with geometries and features closer to the in vivo organ functional units. They spontaneously develop from stem cells, organ‐specific progenitors, and even tumor cells through a self‐organization process.^[^ ^24^ ^]^ Contrarily to spheroids that are usually free‐floating, organoids are often maintained within an ECM structure that offers mechanical support to the cells.^[^ ^25^ ^]^ Thanks to these characteristics, organoids can be exploited as an effective platform not only to evaluate drug response and efficiency, but also to develop personalized medicine approaches^[^ ^26^ ^]^ or study brain aging phenomena in vitro.^[^ ^27^ ^]^ A variety of NDD models have been developed using spheroid or organoid technologies and were able to secure accurate regulation over cell positioning and ECM material composition. Despite all these advantages, they are still at an embryonic phase and massive efforts need to be directed toward the optimization of organoid generation. In fact, to achieve these goals, several limitations have to be overcome,^[^ ^25^, ^26^, ^27^, ^28^ ^]^ including: i) lack of standardization of complex protocols; ii) definition of growth factors and metabolites necessary to prolong the lifespan and self‐renewal of the organoids; iii) high costs and time consumption; iv) lack of vascularization, oxygen, and nutrient diffusion in the center of the organoid; and v) difficulties in replicating age‐related diseases.

Cell source represents the most challenging aspect in the design of a physiologically relevant in vitro model. Cells obtained from animals often face the same disadvantages previously discussed for in vivo models. One possibility is represented by the use of human cell lines that are derived from tumors, human tissues, or body fluids and have become immortalized. These cells can be manipulated and grown easily, and can provide a large amount of cells with limited variability among different studies.^[^ ^29^ ^]^ However, they can be physiologically far from the cells they originated from. For this reason, primary cell cultures are often preferred since they have not been manipulated and, therefore, they can produce highly reliable and physiologically relevant results. However, the tricky aspect of using primary cells is represented by the preparation and subsequent culture. In the neuroscience field, the use of primary cells is more complicated due to two other aspects: i) mature neurons do not proliferate; ii) the quantity of samples derived from biopsies is very limited and sometimes even impossible. Moreover, for the study of NDDs, it is almost impossible to measure the disease progression since multiple sample collections across time points pre‐ and post‐diagnosis are difficult. In addition, ethical concerns limit the collection and use of human brains postmortem for research purposes. In the case human tissues are collected, these have limited usage, being highly susceptible to degradation, and present an immature immunological phenotype.^[^ ^30^ ^]^ Stem cells (SCs) represent a unique opportunity for the study of NDDs and the development of regenerative and personalized medicine approaches, and they could be used for many degenerative diseases where one cell population or part of an organ fails.^[^ ^30^, ^31^ ^]^ Adult stem cells have demonstrated potential for regenerative medicine and, among the different types, the most studied are hematopoietic stem cells. Other SCs that have been widely studied are cardiac stem cells, mesenchymal stem cells, and stem cells derived from the umbilical cord, placenta, and amniotic fluid. Although these cells have useful properties, they are hard to obtain since they are present in low amounts and have limited differentiation potential. An alternative to adult stem cells is represented by human induced pluripotent stem cells (hiPSCs), quickly proposed, after their discovery in 2006, as the major tool in the development of personalized medicine.^[^ ^32^ ^]^ They can be derived from human subjects and differentiated toward specific cell types, even those difficult to obtain from living humans (i.e., motor neurons). Also in this case, there are some limitations in the use of iPSCs technology: not only the lack of standardized protocols, which significantly affects reproducibility, but also the lack of epigenetic factors and age‐related markers, lost during the different steps of genome editing, that are typical of neurodegenerative diseases (i.e., AD or ALS).^[^ ^33^ ^]^

Rising technologies in the field of microfabrication and biomaterials science offer the possibility to overcome the problems related to 2D in vitro and animal models, demonstrating great potential in the replication of diseases and drug discovery. Here we provided an overview on the evolution of technology‐driven in vitro models of neurodegenerative disease. We first summarized the advantages of microfluidics and the main microfabrication techniques used to develop organ‐on‐chip (OoC) systems, which represent an innovative strategy to recapitulate the key functions of one or more organs in a highly compact and physiological manner. An insight into OoC platforms developed for the study of NDDs was later provided. Then, we introduced hydrogel‐based (3D) in vitro culture systems by giving first fundamental details on the types and properties of hydrogels, secondarily providing an extensive synopsis on the design of bioinks and bioprinting approaches for in vitro modeling of NDDs. In the following section, we analyzed 3D microfluidic devices where microfluidics and biomaterials are combined to better recapitulate in vivo conditions, focusing our attention on the models that reproduce the NMJ. Challenges that need to be overcome to bring technology‐driven in vitro models to the next level of physiological fidelity and potential are described in the last Section.

## 2 Microfabrication Technologies for Organ‐on‐Chip Platforms

In 2006, G. Whitesides referred to microfluidics as the science that studies, manipulates, and controls tiny amount of liquid (10^−9^–10^−18^ L) inside continuously perfused, micrometer‐size chambers (from a few micrometers to hundred ones).^[^ ^34^ ^]^ Microfluidic devices, depending on their application, are known as microreactors,^[^ ^35^ ^]^ lab‐on‐a‐chip,^[^ ^36^ ^]^ and OoC.^[^ ^37^ ^]^ Although OoCs share the same fabrication techniques, they have specific characteristics aimed at mimicking essential structural and functional aspects of organ or tissue units. These systems are configured in a user‐friendly format to facilitate extended analysis and manipulation, thus facilitating high‐throughput screening processes. The power of OoC technology lies in the possibility to design ad hoc culture systems in which many parameters, such as types and positions of cells, concentration gradient, oxygen level, and shear stress, can be controlled.^[^ ^38^ ^]^ The primary goal of OoCs is to offer standardized, cost‐effective, yet highly pertinent in vitro models. These models serve dual purposes: advancing the understanding of fundamental biological mechanisms and enhancing the efficiency of drug discovery. By utilizing patient‐derived primary or iPSCs, and their derivatives as cell sources, OoCs have the potential to replicate an individual's disease on a chip. This capability holds promise for diagnostic and therapeutic testing, making OoC systems crucial tools in the realm of personalized and precision medicine therapies. Although in vivo experiments remain the intermediate steps between bench and human body, the need to develop new approaches starts from three considerations: i) first of all, as it has been calculated recently, the total cost for bringing a new drug to the market has been increasing over the years (from $1.1 billion in 2003 to $2.8 billion in 2013);^[^ ^39^ ^]^ ii) second, many drug candidates are withdrawn due to toxicity effects that are visible just in the clinical studies or even when the drug is already in the market. This is strictly related to the poor predictive power of the current in vitro testing platforms, unable to generate reliable prediction data of the drug efficacy and safety in humans and the failure of animal studies to successfully forecast the efficacy and toxicity of new compounds in humans: animal models can recapitulate the human diseases only to some extent, providing a limited amount of human‐relevant data essential in the discovery and development of new drugs; and iii) third, increasing ethical concerns regarding the use of animals and the compliance to the 3Rs rules (replace, reduce, refine) have been arisen in the last few years.^[^ ^40^ ^]^ The OoC technology represents a unique technology for i) reducing the costs associated with preclinical studies; ii) developing physiologically relevant models that can effectively study drug efficacy and toxicity; and iii) respecting the 3R principles by using patient‐derived cells.

### 2.1 Optical and Soft Lithography

At the beginning of 1990s, photolithography was the main technique used to fabricate microfluidic devices in glass and silicon.^[^ ^41^ ^]^ Photolithography utilizes UV light to transfer a desired pattern, present on a hard mask, onto a light‐sensitive polymer called photoresist which is able to change, after the UV exposure, its own chemical structure, resulting in a change in solubility. Despite the high resolution of the single features obtained with this process, high cost, long processing times, specialized and sophisticated capabilities are required, representing an important block to the implementation of photolithography in fast and cheap prototyping of microfabricated materials. Soft lithography overcomes these challenges. Introduced for the first time in 1998 by Xia and Whitesides, soft lithography includes different techniques (replica molding, contact printing, and embossing) that present the shared characteristic of using a patterned elastomer as the mask, stamp, or mold.^[^ ^42^ ^]^

This technique is a two‐step procedure, since two main processes are required: i) a photolithography process in order to fabricate the stamp; ii) the molding process to create the final device. A wide range of materials can be used to realize microfluidic devices^[^ ^43^ ^]^ (i.e., PMMA, polycarbonate, polystyrene, and cyclic olefin copolymers), but one of the most used materials is polydimethylsiloxane (PDMS). In addition to the low cost and the easy casting procedure,^[^ ^44^ ^]^ PDMS has excellent chemical and physical properties:^[^ ^45^ ^]^ i) biocompatibility; ii) optical transparency (down to about 300 nm); iii) gas permeability; iv) chemical inertness; and v) thermal stability. Regardless of these advantages, some physicochemical features of PDMS represent a drawback for particular biological applications. In fact, it is an intrinsically hydrophobic material, and it is able to absorb small molecules making difficult to quantitively analyze experiments in drug discovery and cell culture.^[^ ^46^ ^]^

#### 2.1.1 2D Microfluidic Platform for Neurodegenerative Diseases

**Figure 1:**
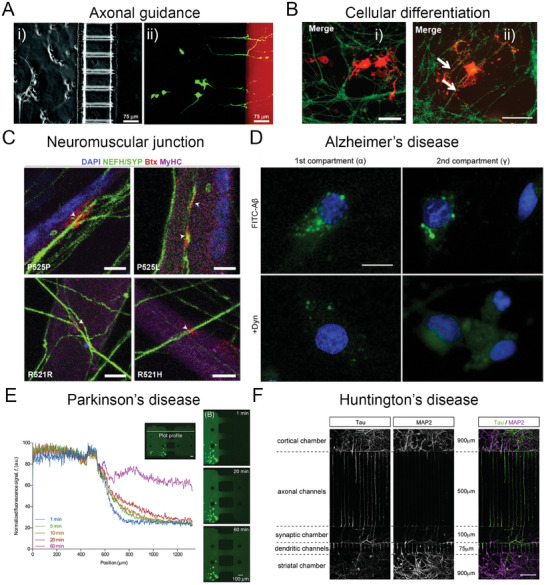
Brain‐on‐chip models. A) Bright‐field (i) and fluorescence (ii) optical micrographs of a two‐compartment system that permits unidirectional growth of neurites and neurite/body confinement, exploiting the introduction of a positive hydrostatic pressure from the soma chamber (on the left of each panel), where neurons are hosted, and the neuritic chamber (on the right), where neurites extend (calcein AM in green, Texas Red dextran in red). Reproduced with permission,^[^ ^50^ ^]^ Copyright 2003, American Chemical Society. B) Differentiation of OPCs and myelin formation were obtained through optogenetic stimulation in a two‐compartment device (ChR2‐EYFP, neurons; MBP, glia). Reproduced with permission,^[^ ^57^ ^]^ Copyright 2016, American Chemical Society. C) NMJ model was realized co‐culturing iPSCs‐derived MNs and human primary mesoangioblast‐derived myotubes. The effect of FUS mutation on the formation of NMJ was evaluated, using ALS‐FUS (P525L, R521H) and isogenic control (P525P, R521R) cocultures. Data showed a decrease in the overall number of junctions, approaching a 50% reduction in the FUS‐P525L model in respect of the control. Reproduced with permission,^[^ ^73^ ^]^ Copyright 2020, Elsevier. D) The transport of extracellular FITC–monomer A*β* was investigated by Song et al in a three‐compartment device: they observed that A*β* adhered to the membrane of distal axons in one chamber, transferred to the cell bodies, then internalized, metabolized, secreted, and further transported to neighboring neurons in another chamber. Reproduced with permission,^[^ ^63^ ^]^ Copyright 2014, John Wiley and Sons. E) Fernandes et al., created a Parkinson's model utilizing pneumatic valves. These were essential to investigate the release and spreading of *α*‐syn, shedding light on its pathogenic potential. Reproduced under the terms of the CC BY 3.0 license,^[^ ^67^ ^]^ Copyright 2016, Frontiers. F) Resembling a corticostriatal circuit‐on‐a‐Chip revealed the effect of the presynaptic compartment in HD. Reproduced with permission,^[^ ^71^ ^]^ Copyright 2018, Elsevier.

By taking advantage of these aspects, microfluidic technologies have been largely applied to the neuroscience field for the study of different phenomena, that is, axonal guidance, neurodegeneration, synapse formation, and cell–cell communication (**Figure** **1**).^[^ ^47^ ^]^ Since the early seventies, the possibility to compartmentalize neuronal cells or cell portions has been eagerly pursued, and Robert Campenot can be considered the pioneer in this context. He developed a compartmentalized device in order to separate neuronal axons from their soma, allowing the study of different neuronal phenomena (i.e., axonal transport and migration).^[^ ^48^ ^]^ Despite the innovative aspect, this device had numerous shortcomings, including the complicated fabrication process and difficulty of use, poor adhesion, and disorders of neurites.^[^ ^49^ ^]^ But it is Taylor's work, in 2003, that represents a landmark for the study of neuronal cell culture and axon biology mechanisms in vitro.^[^ ^50^ ^]^ Taylor and coworkers proposed a two‐compartment microfluidic device in which cells were plated in the microfluidic compartments (having width and height in the order of hundreds of µm), separated by a physical barrier represented by a number of smaller microchannels (having height in the order of a few µm), allowing the growth of neurites across the compartments and simultaneously preserving their fluidic confinement. Since that time, several examples have been provided with various modifications of the layout: the introduction of a third compartment,^[^ ^51^ ^]^ a circular layout,^[^ ^52^ ^]^ and asymmetrical microchannels.^[^ ^53^ ^]^

Microfluidic platforms, due to their versatility, are used to study different aspects of neuroscience. In vivo, a polarized neuron extends its axon across the ECM to connect to the target. This directional neuronal connectivity, that allows the optimization of the speed of processing, is critically involved in neurodegeneration.^[^ ^54^ ^]^ Several microfluidic platforms based on axonal guidance and outgrowth have been successfully used with both human and rodent cells. Peyrin et al., developed a two‐compartment microfluidic platform separated by arrays of rectangular microchannels of decreasing width, called “axonal diodes,” that were able to impose unidirectional axon connectivity.^[^ ^53a^ ^]^ Due to this configuration, they were able to create and characterize a cortico–striatal oriented network. Fantuzzo et al. developed a five‐compartment neurocircuitry as a simplified version of human brain circuitry capable of receiving multiple inputs, with different neuron subtypes derived from patient‐specific hiPSCs.^[^ ^55^ ^]^ Neuronal growth and formation of connections among neurons are also led by multiple guidance cues that allow neurons to reach their target. Most of the important signals are molecular gradients (i.e., neurotrophic factors) that cannot be reproduced using conventional 2D cell cultures. Microfluidic devices, due to their characteristics, allow spatio‐temporal fluid control. For example, Huang et al. evaluated the effect of brain‐derived neurotrophic factor (BDNF) on axon guidance using a serpentine‐based device. They observed a double effect of BDNF on hippocampal neurons: it affected the survival of the neurons also promoting the nerve growth cone guidance.^[^ ^56^ ^]^

The possibility to co‐culture various cell types in different cell compartments, keeping separated media and specific factors, allows to reproduce the interaction between neurons and other cells, such as glia or muscle cells, and study several phenomena (i.e., myelination or demyelination) and functional units (i.e., neuromuscular junction, NMJ) damaged in neurodegenerative disorders, including ALS. Lee et al. co‐cultured mouse neurons and oligodendrocytes precursor cells (OPCs) in a two‐chamber device. They induced the differentiation of OPCs through optogenetic stimulation and observed that both region‐specific and whole‐cell stimulation resulted in the differentiation of OPCs and myelin formation.^[^ ^57^ ^]^ This study pointed out two main aspects. On one hand, optogenetic technology is a new versatile tool that allows precise spatial and temporal control of specific cell populations and is characterized by high precision and minimally invasive nature. It represents, therefore, a promising means of regulating neural fate and understanding the molecular mechanisms underlying neurological diseases.^[^ ^58^ ^]^ On the other hand, the possibility to reproduce the myelin sheath allows to develop a more physiological model. Santahnam et al., using a two‐chamber device, investigated the NMJ functionality in response to increasing doses of three synapse‐blocking molecules: curare, botox, and α‐BTX.^[^ ^59^ ^]^ They demonstrated that motoneurons (MNs)‐triggered myotube contraction is constantly decreased and finally stopped at the highest dose of each toxin. Dittlau et al. generated a co‐culture of iPSCs‐derived MNs and human primary mesoangioblast‐derived myotubes resulting in the formation of a functional NMJ.^[^ ^60^ ^]^ In particular, they observed an increase in NMJ formation following the addition of agrin and laminin since it has been shown that they increase the acetylcholine receptor clustering at the sarcolemma, enhancing the NMJ formation. Moreover, they studied the effect of FUS mutation on the formation of NMJ. They recorded a decrease in the total number of junctions, approaching a 50% reduction in the FUS‐P525L system with respect to the control. In a second work, Dittlau et al. implemented their model integrating iPSCs‐derived astrocytes and evaluating their role on hiPSC‐derived MN network and NMJs functionality.^[^ ^61^ ^]^ They demonstrated that astrocytes have a key role in ALS pathological mechanisms, being associated with destruction of MN network and interfering with NMJ formation and functionality through several gain‐of‐toxicity and loss‐of‐support processes. In a recent work, Fornetti et al. tried to reproduce a reliable NMJ model in vitro able to investigate deficiencies in the neural and muscular cross‐talk behind the junction formation in *α*‐sarcoglycanopathy (*α*‐SG), a subtype of limb‐girdle muscular dystrophy.^[^ ^62^ ^]^ They co‐cultured in a two‐compartment device hiPSCs‐derived MNs and immortalized myoblasts from either healthy donors or *α*‐SG–affected patients to unveil the importance of the cellular components in the junction formation and functioning. Results showed that, while MNs did not affect the muscular differentiation process, vice versa muscle cells showed a significant role in the attraction and maturation of axons. This was highlighted in the presence of *α*‐SG myotubes, where axon growth across the microchannels was severely affected.

Damage in the neural circuits is responsible for motor dysregulation and cognitive impairment. Due to its complex structures, in vivo or in vitro studies are not easy to perform. Microfluidic platforms can be powerful for the design of in vitro physiologically and pathologically relevant models. Many efforts have been done primarily on Alzheimer's disease (AD), Parkinson's disease (PD), multiple sclerosis (MS), and Huntington's disease (HD).

Even though immune response is an important factor in the pathogenesis of AD, aberrant accumulation of amyloid‐*β* (A*β*) and pTau proteins, and neurodegeneration are considered to be the major hallmarks of the disease. Using a three‐compartment microfluidic device, Song et al. showed that extracellular A*β* was absorbed to the cell membranes of distal axons and transferred to their soma, where the proteins are internalized, metabolized, and secreted, reaching neighboring neurons.^[^ ^63^ ^]^ Deleglise et al. used a two‐compartment microfluidic platform to recreate an in vitro cortico–striatal neuronal network model and investigate the toxic effect of A*β*. Adding A*β* peptide to the neurites channel, they observed a dying‐back process in the soma compartment.^[^ ^64^ ^]^ Katsikoudi et al. realized a model for human sporadic tauopathies using primary non‐transgenic rat cortical neurons and human AD (hAD) seeds (self‐replicating assemblies) extracted from AD patient's brains.^[^ ^65^ ^]^ They observed that hAD seeds induce clustering of endogenous Tau in the neurons as well as their propagation. Then, they tested a molecule (di‐phenyl‐pyrazole anle138b) known to impede A*β* aggregation in vitro and in vivo. Data showed that the addition of this compound decreased the formation of neuritic thread‐like protein inclusions and, as a consequence, the number of propagated inclusions. In a study by Nobuhara et al., some Tau antibodies (i.e., Tau13, 6C5, HT7), targeting the protein mid‐domain, were able to effectively inhibit the uptake of Tau proteins and subsequent aggregation and propagation.^[^ ^66^ ^]^

Parkinson's disease shows distinctive decrease of dopaminergic neurons localized in the substantia nigra and modulation of the deposit of intracellular protein inclusions, in particular alpha‐synuclein (*α*‐syn), known as Lewy bodies. For this reason, understanding *α*‐syn formation and trafficking is crucial. Fernandes et al. proposed a two‐compartment device with integrated pneumatic pumps that allowed the temporal control of cell treatment and diffusion between compartments.^[^ ^67^ ^]^ They investigated two different phenomena: i) the release and spreading of GFP‐tagged *α*‐syn using naïve H4 neuroglioma cells; ii) a higher formation of reactive oxygen species in H4 cells co‐cultured with activated N9 microglia cells. Gribaudo et al. resembled in microfluidic systems cortico‐cortical neuronal circuits using hiPSCs derived from a healthy donor, and investigated the effects of two distinct *α*‐syn forms, named fibrils and ribbons, on neuronal behavior.^[^ ^68^ ^]^ They found that both these forms were transported in the healthy neurons in a “prion‐like” manner, resulting in a progressive accumulation dose‐ and structure‐dependent. Moreover, these aggregates disrupted synaptic integrity and mitochondria morphology. Volpicelli–Daley et al. investigated whether direct addition of α‐syn pre‐formed fibrils to either neurites or soma is correlated to spreading of pathologic *α*‐syn aggregates throughout the neuron.^[^ ^69^ ^]^ They observed that the accumulation of these aggregates is connected to severely damaged synaptic connections compromising neuronal excitability.

Multiple sclerosis is classified as a demyelinating disorder, in which axons are damaged during acute demyelination process. Licht–Mayer et al. proposed a demyelination model using an axonal guidance device in which neurons and oligodendrocyte precursor cells were co‐cultured.^[^ ^70^ ^]^ They induced demyelination by exposing the axonal compartment to lysolecithin and investigated a new mechanism in which mitochondria shift from the cell body to the demyelinated axon, thus incrementing the number of local mitochondria. This phenomenon seems to prevent acute degeneration in demyelinated axons.

In Huntington's disease, the main deficit occurs at the level of corticostriatal circuit. So far, very few models have been developed to investigate the molecular mechanism of this pathology and test potential drug candidates and the OoCs technology could represent a suitable means in this regard. Virlogeux et al. tried to reproduce an HD corticostriatal network using a two‐compartment platform.^[^ ^71^ ^]^ They identified frequent pre‐ and postsynaptic modifications that lead to dysfunctions and hypersynchrony of the entire circuit. Moreover, they demonstrated that the genetic condition of the presynaptic element affects the circuit integrity. Lenoir et al. used a microfluidic device to reproduce an HD corticostriatal network to investigate the pathogenic mechanism and drug activity of pridopidine.^[^ ^72^ ^]^ They demonstrated that this drug is able to augment the transfer of BDNF from the cortex to the striatum, recover the synapse homeostasis, and the BDNF‐receptor dynamics within the striatum.

### 2.2 3D Printing

**Figure 2:**
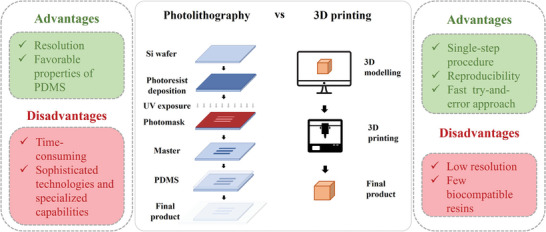
Classic lithography and 3D printing techniques. Overview of advantages and disadvantages of both classic lithography techniques and 3D printing ones.

Albeit microfluidic devices can recapitulate biochemical and biomechanical cues and are characterized by high efficiency, replicability, and controllability, two main drawbacks prevent their use on a large scale in biological research. First, the OoC traditional production is time‐consuming, and specialized capabilities and sophisticated technologies are required. Moreover, plain microfluidic devices cannot mimic the natural structure of ECM in vivo, since a 2D monolayer cell culture is often reproduced. The rising technology of 3D printing, belonging to additive manufacturing (AM) or rapid prototyping, could be used to overcome these downsides.^[^ ^74^ ^]^ 3D printing approaches offer the possibility, from one side, to increase the thickness of the microfabricated devices, and, moreover, to fabricate the whole device in a single step, reducing time and costs, which means that the final structure is obtained, starting from the digital data, using a single machine^[^ ^75^ ^]^ (**Figure** **2**). The 3D printing process is composed of two main procedures: i) the design modeling using a CAD or other drawing software; and ii) the object production.^[^ ^76^ ^]^ Once the device is fabricated, it may undergo further physical and chemical treatments (i.e., cleaning, UV curing, and plasma treatment) to modify or clean up the surface. Not all the 3D printing techniques are suitable for microfluidic device fabrication. The most popular in this field are laminated object manufacturing and stereolithography (SL), although other approaches such as selective laser sintering, VAT photopolymerization, fused deposition modeling, and photopolymer inkjet printing can be used adding assembly steps.^[^ ^77^ ^]^ The popularity of 3D‐printed microfluidic devices is, however, still limited. This is due to two main shortcomings: i) the resolution of the manufacturing process is still far from that obtained with lithography techniques; and ii) most of the resins and materials used in 3D printing do not have the exceptional properties of PDMS. Consequently, until now, most of the 3D microfluidic devices for the study of neurodegeneration processes are still realized using classical lithography techniques and the 3D component (often referred as 2.5D) is approximately represented by the presence of thin layer of hydrogel (i.e., matrigel or collagen) in which a few layers of cells are embedded.^[^ ^78^ ^]^ In the very recent years, commercial two‐photon 3D‐printing systems have been developed to overcome the issues related to the insufficient resolution of the common 3D printing technology. Two‐photon polymerization (2PP) can be considered as equivalent for 3D printing on the micron scale. Contrarily to UV lithography and 3D printing via stereolithography, 2PP treats the resin with femtosecond laser pulses in the visible to near‐infrared spectral region.^[^ ^79^ ^]^ Thanks to the high resolution (<1 µm), the use of this technology is spreading for the fabrication of bio‐microdevices and bio‐nanodevices. However, the application in neurobiology research is still limited. Harberts et al. fabricated a 3D nanoprinted microscaffold via 2PP technology to guide neuronal growth and, employing human iPSCs differentiated into neurons, to assess the maturation of a functional neuronal circuit within the microfabricated architectures.^[^ ^80^ ^]^

## 3 Hydrogel Systems and Bioinks for the In Vitro Modeling of 3D Microenvironment

Most of the current knowledge about biological phenomena comes from experiments conducted in classical cell cultures on 2D substrates. It is now well‐established, however, that the results obtained with this type of platform are too reductive to be accurately translated to the human body. In 2D substrates, cells indeed live in an environment that differs significantly from the 3D one present in vivo, so their growth is also different: tissue‐specific polarity is absent, cell–cell communication by direct contact is limited, non‐physiologically (uniform) diffusion kinetics are present. These features modify the way cells interact with the surrounding microenvironment. 3D culture systems, such as those based on hydrogels, which are able to more accurately mimic the native in vivo environment, represent a promising strategy in this regard. Indeed, the 3D microenvironment can heavily affect cell fate and functions, becoming an important aspect in all the different cellular mechanisms, from cell proliferation to differentiation, migration, and death.

### 3.1 Hydrogels: Properties and Classifications

In the wide range of biomaterials used for 3D systems, polymers play a fundamental role due to their great flexibility.^[^ ^81^ ^]^ In this regard, hydrogels receive particular attention. They are referred to as “a 3D hydrophilic polymer network, capable of absorbing and retaining large amounts of water or biological fluids.”^[^ ^82^ ^]^ They are characterized by unique physical properties such as degradability, gelation time, and temperature, mechanical properties, and degrees of swelling, which make them widely tunable and suitable for various applications, such as controlled drug delivery systems, medical devices, as well as cell encapsulation systems in 3D cell cultures.^[^ ^83^ ^]^ In addition, a number of chemical modifications can be designed to make hydrogel materials “smart,” that is, capable of responding to particular external stimuli, such as temperature, light, pH, and ion concentration, with specific effects.^[^ ^84^ ^]^ In particular, due to their properties and features, they are suitable as ECM analogous in 3D culture systems by faithfully reproducing chemical and physical characteristics of the native environment.^[^ ^85^ ^]^ Hydrogels can be classified in different ways depending on their origin, mechanical properties, and crosslink strategy.

#### 3.1.1 Origin: Natural and Synthetic Polymers

Polymers of natural origin show higher biocompatibility, biodegradability, and better interaction with cells due to their similarity with ECM characteristics, but they often suffer in terms of tunability of mechanical properties, that are usually low due to their intrinsic nature.^[^ ^86^ ^]^ Natural polymers include a wide variety of materials, such as silk fibroin, collagen, gelatin, hyaluronic acid, chitin and chitosan, and alginate. Silk fibroin is a fibrous protein that is used extensively in tissue engineering for the formation of hydrogels and scaffolds. Silk fibroin‐based hydrogels can be obtained modulating parameters such as pH, shear stress, or osmolarity that modifying the hydrophobic regions allow the formation of hydrogels.^[^ ^87^ ^]^ Collagen is a constitutive element of the ECM and in vivo displays structural functions, but it is also involved in cell processes, such as proliferation and differentiation.^[^ ^88^ ^]^ The use of collagen for the formation of hydrogels is widely popular in 3D culture systems, with optimal results in terms of interaction with cells.^[^ ^89^ ^]^ Gelatin is a polymer derived from the hydrolysis of collagen, highly biocompatible and biodegradable. It possesses the advantage of being easily soluble in aqueous solutions in addition to being available at low cost, making it more advantageous than collagen from which is derived.^[^ ^90^ ^]^ Hyaluronic acid is a polysaccharide containing repeated disaccharide units of *N*‐acetyl‐d‐glucosamine and d‐glucuronic acid. Hyaluronic acid is a natural and ubiquitous component of the ECM of many body districts and therefore finds high applicability in the use of 3D culture systems.^[^ ^91^ ^]^ Thanks to its high biocompatibility and the presence of reactive groups in its structure, its chain can be easily modified to prepare hydrogel systems for tissue engineering.^[^ ^92^ ^]^ Chitin is a polymer extracted from the exoskeleton of crustaceans from which chitosan, the deacetylated form of chitin, is derived. Both polymers are widely used in the biomedical field as they are characterized by high biocompatibility, biodegradability, low toxicity, adhesive properties, and antibacterial properties.^[^ ^93^ ^]^ Alginate is an algae‐derived polymer, readily available at low cost and one of the most widely used polymers for 3D culture systems. Alginate hydrogels are crosslinked in various ways and mimic many of the natural characteristics of the ECM. This polymer exhibits strong biocompatibility, low toxicity, and biodegradability properties.^[^ ^94^ ^]^

Synthetic polymers display a great benefit over natural polymers: they have better mechanical properties and stability, can be modified in an efficacious and simple manner, and present low batch‐to‐batch variability. On the other hand, they show, in many cases, poor biocompatibility.^[^ ^86^ ^]^ Poly(*N*‐isopropyl acrylamide) is a synthetic polymer with hydrophilic and hydrophobic functionality, one of the most used to form hydrogels. It has the advantage of performing the sol–gel transition at temperature values just below body temperature, around 32 °C, which makes it widely used for biomedical applications and in 3D culture systems, where it shows high interaction with cells and involvement in differentiation processes.^[^ ^95^ ^]^ Another synthetic polymer widely used in the biomedical field is poly(ethylene glycol) (PEG), which has the great advantage of possessing high biocompatibility, degradability, and low immunogenicity and toxicity. Due to its nature, it can be easily modified and therefore finds application in a wide range of fields, such as 3D culture systems, drug delivery, and biomedical systems.^[^ ^96^ ^]^ Polyurethane is a synthetic polymer consisting of hydroxyl and isothiocyanate functionalities that enhance the adhesive capabilities of this polymer. Polyurethane exhibits strong mechanical properties and in hydrogel form has a characteristic porous structure that makes it suitable for 3D culture systems, facilitating the transport of nutrients and catabolites.^[^ ^97^ ^]^

#### 3.1.2 Crosslinking

Crosslinking of hydrogels can occur according to two different routes: chemical or physical. The type of crosslinking strongly influences the structure and downstream properties of the hydrogels. Chemical crosslinking is the most used strategy. It consists in the formation of strong and irreversible chemical bonds between polymer links.^[^ ^98^ ^]^ Chemical crosslinking can be performed in a variety of ways including covalent bond formation,^[^ ^99^ ^]^ chemical coupling,^[^ ^100^ ^]^ or click reactions.^[^ ^101^ ^]^ Chemically crosslinked hydrogels commonly exhibit improved stability, better mechanical properties, and controllable degradability.^[^ ^102^ ^]^ Chemical crosslinking which can be accomplished by specific chemical reactions can be modulated to obtain a hydrogel with highly tunable properties based on the degree of crosslinking.^[^ ^103^ ^]^ On the other hand, physical crosslinking results in the formation of weak intermolecular bonds that are reversible. They generally consist of electrostatic interactions, hydrogen bonds, and weak interactions.^[^ ^98^ ^]^ The advantage of not having a permanent crosslinking confers to these hydrogels the possibility of self‐regeneration and to be easily injectable especially at room temperature. For these reasons, they are widely used in the biomedical field. In addition, the lack of chemical modification allows them to retain their original biocompatibility.^[^ ^104^ ^]^ Another type of crosslinking is based on the dynamic covalent chemical bonds. In this type of crosslinking, there is a constant balance between products and reactants.^[^ ^105^ ^]^ The dynamic covalent hydrogels show a viscoelastic behavior.^[^ ^106^ ^]^ Unlike traditional hydrogels, in which crosslinking bonds are generally covalent and permanent, dynamic crosslinker hydrogels are characterized by reversible bonds that can break and reform in response to external stimuli.^[^ ^107^ ^]^ These bonds can be weak and reversible (e.g., as in the case of hydrogen bonds), allowing the hydrogel to exhibit dynamic properties such as the ability to regenerate, adapt, and self‐heal.^[^ ^108^ ^]^ The choice of the type of crosslinking, in addition to defining the type of hydrogel and its stability, significantly influences its mechanical properties which are fundamental when the hydrogel is applied in 3D culture systems such as ECM.

The mechanical properties of hydrogels are poor due to the nature of the system itself. The high tunability of hydrogels, however, has allowed in recent years the implementation of strategies aimed at improving the mechanical strength of hydrogels to the point that these can now meet the mechanical requirements of several target tissues.^[^ ^109^ ^]^ The mechanical properties can be modulated by acting on the crosslinking of the material in terms of type or degree of crosslinking, which influences the stiffness of the material.^[^ ^110^ ^]^ The parameter that is usually evaluated to assess the mechanical properties of hydrogels is the Young's modulus at compression or tension, also called compressive or tensile modulus of elasticity, which quantifies the rigidity of a material in compression or traction, when it undergoes a uniaxial load.

#### 3.1.3 Responsivity

Another criterion for classifying hydrogels, especially those defined as “smart,” is the responsiveness to different stimuli. The most frequent stimuli that can induce certain responses in hydrogels are pH, temperature, light, or a mechanical stimulus. The pH responsiveness of hydrogels determines their swelling in the presence of acidic or basic environments depending on the presence of reactive groups. The presence of fixed charges on the polymer chains determines the swelling of the hydrogels which can have the maximum responsiveness in the presence of small pH variations.^[^ ^103^ ^]^ Thermoresponsive hydrogels make the sol–gel transition in the presence of a rise or fall in temperature. Widely used in 3D culture systems are hydrogels that perform the sol–gel transition at body temperatures ensuring the viability of the encapsulated cells.^[^ ^111^ ^]^ Another widely used stimulus is UV light, which finds application especially in sophisticated bioprinting systems. The disadvantage of this type of stimulation is that it can induce cellular damage, but controlled exposure is one way to solve this problem.^[^ ^112^ ^]^ Mechano‐responsive hydrogels have recently been investigated and are widely used in controlled drug delivery systems. In this class of hydrogels, the presence of an external or internal mechanical stimulation leads the hydrogel to an internal response, consisting of a remodulation, disruption, or creation of chemical interactions, which ends when the applied force is interrupted. This behavior and the remodulation of the chemical interactions of the hydrogel, following the mechanical stimulus, allow, for example, an easier proliferation of the cells it contains.^[^ ^113^ ^]^ Another class of hydrogels responsive to external agents is represented by magnetically responsive hydrogels, capable of responding to external magnetic fields by changing their properties or behavior. These hydrogels usually consist of a polymeric matrix that incorporates magnetic particles, such as iron oxide nanoparticles (for example, magnetite, or hematite), within their structure.^[^ ^114^ ^]^ The inclusion of such magnetic particles allows the hydrogel to respond to applied magnetic fields. Magnetically responsive hydrogels offer multiple opportunities in different fields, such as biomedicine, biotechnology, and robotics. They allow a remote and non‐invasive control of their properties and can be used in various applications, both in vitro and in vivo.^[^ ^115^ ^]^

### 3.2 Cell‐Laden Hydrogels as 3D Neuronal Culture Systems

**Figure 3:**
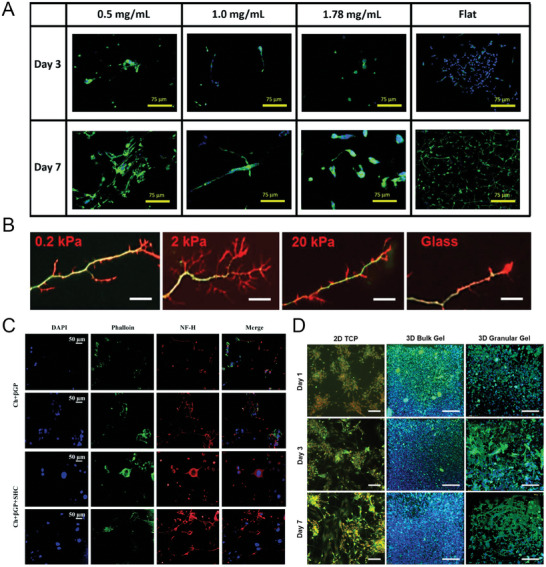
Overview of hydrogels in 3D neuronal culture systems. A) SH‐SY5Y cells encapsulated in hydrogels with different collagen concentrations and in 2D (Flat), visualized under immunofluorescent microscopy. Green = *β*3‐tubulin, blue = DAPI. Reproduced under the terms of the CC BY 4.0 license,^[^ ^119^ ^]^ Copyright 2021, Royal Society of Chemistry. B) Hydrogels composed of methylsulfone acrylate, acrylamide, and acrylic acid, and biofunctionalized with laminin peptide (IKVAV) and poly‐lysine displaying different stiffness were used to culture neuronal progenitor cells: the hydrogel with a stiffness of 2 kPa showed a noticeable increase in filopodia (in red) along the entire axon (green). Scale bar: 10 µm. Reproduced under the terms of the CC BY 4.0 license,^[^ ^121^ ^]^ Copyright 2017, Elsevier. C) Immunofluorescence observation of NSC‐34 cells laden chitosan‐based hydrogels at 8 days of differentiation (DAPI for nuclei, phalloidin for actin, and heavy non‐phosphorylated neurofilament for neurites) showing an elongated shape and the presence of neurites. Reproduced with permission,^[^ ^120a^ ^]^ Copyright 2021, Royal Society of Chemistry. D) Visualization of viability up to day 7 of NPCs in granular or homogeneous (bulk) hydrogels and tissue culture plate (TCP). Signals refer to calcein AM, green; DRAQ5, red/blue; scale bars = 100 µm. Reproduced under the terms of the CC BY 4.0 license,^[^ ^125^ ^]^ Copyright 2022, Elsevier.

The brain is certainly one of the most complicated architectures to reproduce in vitro because of its composition and structural complexity. A winning strategy could be the use of hydrogels taking advantage of their biocompatibility, great versatility, and tunability in composition, stiffness, functionalization, etc.^[^ ^116^ ^]^ In this regard, the literature proposes several in vitro models for NDDs (**Figure** **3**) that employ hydrogel systems both as ECM mimicking structures and drug delivery carriers (**Table** **1**). Preliminary studies, aimed at assessing hydrogels as models for studying neurodegenerative diseases, used immortalized cell lines such as human neuroblastoma (SH‐SY5Y), rat pheochromocytoma, (PC12) or murine motor neuronal‐like cell (NSC34).^[^ ^117^ ^]^ These systems not only confirmed the suitability of hydrogels in the study models, but allowed also to investigate processes, such as cell maturation, proliferation, and differentiation, in a 3D configuration.^[^ ^118^ ^]^ A recent study by Merryweather et al. demonstrated that the mechanical properties of the 3D environment are critical and significantly influence cell morphology. In particular, they showed that even a small variation in the mechanical properties of type I collagen‐based hydrogel, obtained by varying the polymer concentration, influenced both SH‐SY5Y morphology and neurite growth.^[^ ^119^ ^]^ The 3D environment also promotes cell differentiation processes, as reported in a recent study where the length of neurites developed during the differentiation of NSC34 cells was significantly higher for cells encapsulated in a stiffer thermo‐responsive chitosan hydrogel.^[^ ^120^ ^]^ Significant results on the importance of 3D systems in the study of neuronal diseases have been obtained mainly with the use of both neural stem cells (NSCs) and iPSCs. In a study by Farrukh et al., embryonic‐ and adult‐derived neuronal progenitor cells (aNPCs) were encapsulated in hydrogels composed of methylsulfone acrylate, acrylamide, and acrylic acid, biofunctionalized with laminin peptide (IKVAV) and poly‐lysine, where the hydrogel stiffness was modulated. This was tuned in the range of 0.2 and 20 kPa by varying the crosslinker amount (*N*,*N*′‐methylene‐bis‐acrylamide), used for the radical copolymerization reaction.^[^ ^121^ ^]^ Hydrogels showing stiffness typical of brain tissue showed that progenitor cells grew more adherent to the biomaterial and expressed crucial markers in neurite elongation. Tanaka et al. showed that stiffness is not only critical for proper neuronal differentiation, but that inadequate stiffness of the 3D environment can inhibit neurogenesis.^[^ ^122^ ^]^ In their system, hippocampal neuronal cells were tested with polyacrylamide hydrogels with altered stiffness to assess how Young's modulus values beyond the neuronal tissue range can completely impede cell neurogenesis. However, the 3D environment is not only critical for the proper morphological development of neuronal cells but also for functional aspects. Li and colleagues encapsulated neuronal stem cells in 4‐dibenzocyclooctynol (DIBO)‐PEG hydrogel bound with laminin and interferon‐*γ*, demonstrating that they did not exhibit adaptive morphology, although preserving functionality.^[^ ^123^ ^]^ In a recent study, Nazari et al. demonstrated efficient oligodendrocytic differentiation of iPSCs in a fibrin‐based hydrogel and showed that the proliferation rate of cells was significantly higher than in monolayer culture.^[^ ^124^ ^]^ In another very recent work by Hsu and colleagues, a granular laminin‐based hydrogel was developed in which human cortical iPSCs showed high viability and good morphology for up to 7 days of culture.^[^ ^125^ ^]^ Fiore and colleagues observed how 3D platforms are highly informative in reproducing aberrant phenotypes of disease models.^[^ ^126^ ^]^ Specifically, iPSC‐derived neurons, from a court of healthy donors and PD patients with specific known mutations, were cultured in 2D and silk‐based 3D systems. Neurons from both patients and healthy individuals showed not only better dopaminergic network development in the 3D environment, but also a higher rate of *α*‐synuclein deposition, effectively mimicking the situation that occurs in vivo. Hydrogels are also efficiently used in the treatment of neurodegenerative diseases as drug delivery systems. In PD, the administration of l‐dopa is the most pursued strategy to obtain a significant effect. In this case, the administration of l‐dopa is carried out at high doses, due to the low half‐life of the molecule, causing biological resistance. In this context, hydrogels can represent a valid strategy. Wang et al. developed self‐assembled hydrogels capable of effectively conveying the drug in a highly precise manner.^[^ ^127^ ^]^

**Table 1:** Overview of hydrogels used in 3D culture systems for the study of neurodegenerative diseases.

Hydrogel	Cellular Type	Cellular Response	Reference
Collagen	SH‐SY5Y	Elongated morphology and development of neurites	Merryweather et al.^[^ ^119^ ^]^
Chitosan	NSC34	Differentiation into MN‐like phenotype and neuron elongation	Stanzione et al.^[^ ^120a^ ^]^
Methylsulfone acrylate, acrylamide, and acrylic acid functionalized with IKVAV and PL	aNPCs	Adhesion and expression of neuritogenic markers	Farrukh et al.^[^ ^121^ ^]^
Polyacrylamide	Hippocampal neuronal cells	Neuritogenesis	Tanaka et al.^[^ ^122^ ^]^
DIBO‐PEG with Laminin and interferon‐γ	NSCs	Physiological morphology and functionality	Li et al.^[^ ^123^ ^]^
Fibrin	iPSCs	Proliferation	Nazari et al.^[^ ^124^ ^]^
Laminin	iPSCs	High viability and adequate morphology	Hsu et al.^[^ ^125^ ^]^
Silk	iPSCs	Reproduction of the dopaminergic phenotype	Fiore et al.^[^ ^126^ ^]^

The examples mentioned above highlight the importance of using hydrogels for in vitro modeling neurodegenerative diseases. These highly hydrated polymeric materials offer a 3D environment that can simulate the mechanical characteristics of central and peripheral nervous system ECM and allow for the study of pathogenic mechanisms behind, for example, AD, PD, and ALS. Their polymer network can be also functionalized to improve cell adhesion and functioning further or modified to serve as controlled release system for the delivery of drugs directly to the brain or to the regions affected by NDDs.^[^ ^5^ ^]^ An important future step could be developing hydrogel‐based cell therapies, using such systems as a support for the transplantation of stem cells into the brain, providing a 3D environment for the survival, differentiation, and integration of the transplanted cells.^[^ ^128^ ^]^ Furthermore, hydrogels functionalized with contrast agents could find an interesting application in neural imaging and diagnostics, allowing the visualization and analysis of the brain structures affected by the neurodegenerative disease.^[^ ^129^ ^]^

### 3.3 Bioprinting and Bioinks

**Figure 4:**
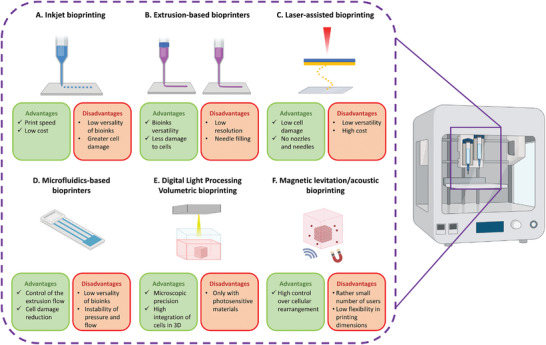
Schematic illustration of six bioprinting methodologies. A) Inkjet bioprinting: in this method the material is extruded as a drop. B) Extrusion‐based bioprinters: the cartridge can work with pressure, piston, or screw, allowing the extraction of the bioink as a filament. C) Laser‐assisted bioprinting: transfer of the material onto a substrate by the action of a laser. D) Microfluidics‐based bioprinters: combined extrusion with microfluidic systems for precise flow control. E) DLP volumetric bioprinting: combination of bioprinting with light projection to achieve fast and accurate printing of biological materials. F) Magnetic levitation/acoustic bioprinting: use of magnetic fields or acoustic waves to manipulate and position cells and biological materials.

One of the most advanced technologies that has been generating high scientific interest in recent years, is represented by 3D bioprinting. This technology allows to print bioinks in order to obtain 3D structures finely adjustable in terms of geometry and simultaneous positioning of different bioinks.^[^ ^130^ ^]^ The 3D bioprinters can be classified into four main types based on the deposition method, that is, inkjet, extrusion, laser‐assisted, and microfluidics‐assisted extrusion bioprinting^[^ ^131^ ^]^ (**Figure** **4**).

#### 3.3.1 Inkjet Bioprinting

In this type of printing, bioink is emitted in the form of droplets deposited on a substrate employing a piezoelectric or thermal system. The benefits of this method are the speed of printing and its low costs. The disadvantages, on the other hand, are related to possible detrimental effects to the cells during deposition, due to temperature or mechanical stress. Another disadvantage is the low variety of materials that can be used since the suitable bioinks must have a low viscosity (typically from 3.5–12 mPa s) and also cannot be loaded with high cell densities.^[^ ^132^ ^]^

#### 3.3.2 Extrusion‐Based Bioprinting

This printing methodology uses pneumatic, piston and screw cartridges. Extrusion‐based bioprinters are in general the most widespread as they are versatile and allow production even on a large scale. The bioink is extruded under constant pressure or speed as a continuous filament.^[^ ^133^ ^]^ The main advantage of this method consists in avoiding stressful conditions for cells, such as heat, shock, and mechanical stress, that are more marked in other types of printing and likely harmful to bioprinted cells. Another important advantage lies in the wide range of bioinks in terms of viscosity and composition that can be successfully used. The principal disadvantages, however, are the low resolution of the printed structures, the frequent clogging of the nozzles and the deformation of the hydrogels in terms of fidelity to the shape of the post‐printed fiber, and collapse of the structures stacked in layers.^[^ ^134^ ^]^

#### 3.3.3 Laser‐Assisted Bioprinting

This technology uses the presence of a laser to deposit a bioink onto a specific substrate. This technique, though very sophisticated and expensive, allows the use of bioinks that have high viscosity or very high cell concentration. Moreover, the inks printed with this method do not undergo the same stresses and perturbations that they suffer when passing through a nozzle, resulting in a low‐risk technique for cells and the absence of nozzle clogging problems. The disadvantages concern, in addition to the high costs, the lack of versatility of the technique, and the choice of materials, as only a few materials are compatible.^[^ ^135^ ^]^

#### 3.3.4 Microfluidics‐Assisted Extrusion Printing

The combination of extrusion and microfluidics technologies has allowed the birth of microfluidics‐assisted extrusion printing. Its great advantage consists in a full control of the flow during the extrusion of the bioink, reducing the shear stress during deposition. The method allows to extrude several types of bioinks at the same time and to finely control the flow parameters, obtaining high‐resolution hollow structures, fibers, or vascularized geometries. On the other hand, the disadvantages include a partial control of material positioning, flow problems, and pressure instability.^[^ ^136^ ^]^

#### 3.3.5 Digital Light Processing Volumetric Bioprinting

Digital light processing (DLP) volumetric bioprinting is an advanced technique that uses digital light projection technology for the 3D fabrication of biomimetic structures. This approach combines bioprinting with light projection to achieve fast and accurate printing of biological materials.^[^ ^137^ ^]^ In this technique, a digital light projector is used to project 2D images onto a volume of photosensitive material.^[^ ^137^ ^]^ The photosensitive material can be a hydrogel containing cells, biomaterials, or other biocompatible substances. The projected light is focused on thin layers of the material, triggering chemical or photo‐induced reactions that lead to polymerization or crosslinking of the material. The main features and advantages of volumetric bioprinting include the speed and precision enabling the production of complex structures with microscopic details, the high resolution and flexibility of materials.^[^ ^138^ ^]^ This technique has promising applications in both regenerative medicine and in vitro disease modeling. The ability to print 3D structures with microscopic precision and the integration of live cells enable the creation of more accurate biological models and the production of functional tissues and organs for research and therapeutic purposes.^[^ ^137^ ^]^

#### 3.3.6 Magnetic Levitation/Acoustic Bioprinting

Magnetic levitation/acoustic bioprinting is an advanced bioprinting technique that uses magnetic fields or acoustic waves to manipulate and position cells and biological materials during the bioprinting process. These methods offer greater precision and control in the 3D arrangement of cells, enabling the creation of high‐quality, complex structures.^[^ ^139^ ^]^ Magnetic levitation bioprinting involves the use of functionalized magnetic particles, which can be incorporated into cells or biological materials to be printed. Using controlled magnetic fields, these magnetic particles can be manipulated and moved to place cells or materials into desired locations.^[^ ^140^, ^141^ ^]^ Acoustic levitation bioprinting, on the other hand, uses acoustic waves instead of magnetic waves during the bioprinting process.^[^ ^142^ ^]^ Acoustic waves generate a force field that allows biological particles to be positioned and manipulated, allowing the formation of defined and precise 3D structures.^[^ ^143^ ^]^

Bioinks are defined as biomaterials processed by bioprinting and incorporating cells and/or molecules and factors.^[^ ^144^ ^]^ To be used as a bioink, a biomaterial must possess specific printability requirements, such as suitable viscoelasticity properties, in situ gelation, maintenance of high post‐print resolution, and high cytocompatibility of the degradation products.^[^ ^145^ ^]^ In particular, the printability of a bioink depends on its composition and its interaction with the printing substrate as well as on the surface tension that is generated.^[^ ^146^ ^]^ The formulation of a good bioink, showing high printing fidelity and high viability of the cells it incorporates, cannot disregard the study of the rheological properties, defining the ink behavior in terms of viscoelasticity, shear‐stress response, and flowability.^[^ ^147^ ^]^ Based on the premises previously described, hydrogels can be good candidates to design novel bioinks. The challenge encountered during the optimization of the printability properties of the hydrogels lies in the modulation of the physicochemical properties in order to have a rapid post‐printing gelation and the maintenance of the 3D shape of the printed construct.^[^ ^148^ ^]^ The major cause of cell damage during printing is shear stress. In this context, the use of a hydrogel formulation as bioink can reduce cell damage. By using low shear‐stress hydrogels and modulating the elastic modulus *G*′ and the viscosity modulus *G*″, that is, tuning the flow behavior, it is possible to obtain non‐Newtonian shear‐thinning materials that preserve cell viability, by screening cells from shear stress.^[^ ^149^ ^]^

### 3.4 Bioprinting for In Vitro Modeling of Neurodegenerative Diseases

**Figure 5:**
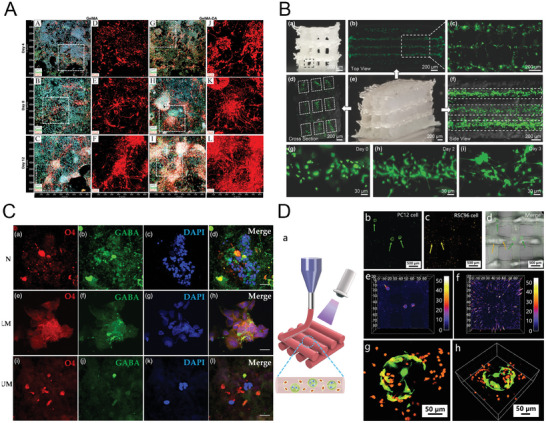
Overview of bioprinted neuronal systems. A) Neural differentiation of NSC cells in GelMA (A–C) and GelMA‐DA (G–I) scaffolds for 4, 8, and 12 days. The analysis was conducted by immunocytochemistry and confocal micrography, acquiring TUJ1 (red), nestin (green), and DAPI (gray) signals. The images (D–F) and (J–L) are an enlargement of the images (A–C) and (G–I) by zooming a specific area. Reproduced with permission,^[^ ^151^ ^]^ Copyright 2018, American Chemical Society. B) Neurocompatibility analysis on alginate‐based scaffolds: a) visualization of a transversal section of the scaffold with channel resolution of ≈150 µm; b–f) visualization of the viability up to day 3 of cells on the scaffolds and in all layers with visualization of the cells from the top (b,c), cross section (d), lateral longitudinal view (f); g–i) visualization of the formation of cellular processes that elongate within the scaffold. Reproduced with permission,^[^ ^152^ ^]^ Copyright 2018, John Wiley and Sons. C) Immunohistochemistry of NPC cells on 3D constructs. The signals refer to O4 (red), GABA (green), and DAPI (blue), scale bar: 20 µm. Reproduced under the terms of the CC BY 4.0 license,^[^ ^154^ ^]^ Copyright 2021, John Wiley and Sons. D) Analysis of PC12 and RSC96 cells embedded in composite structures: a) sketch of the printing process: b–h) confocal fluorescent microscopy images of the two‐layer structures showing PC12 and RSC96 cells, stained with CellTracker Green and CellTracker Orange, respectively. Reproduced with permission,^[^ ^157^ ^]^ Copyright 2020, Elsevier.

Bioprinting technology could be a promising approach to study neurodegenerative diseases (**Figure** **5**), taking advantage of the possibility to replicate highly complex structures, as in the case of brain architecture^[^ ^150^ ^]^ (**Table** **2**). One of the most important biological questions in the study of neurodegenerative diseases is how to recreate neuronal networks. In an interesting work by Zhou et al., the differentiation capacity of 3D printed neuronal stem cells was investigated by bioprinting using a dopamine‐functionalized methacrylate gelatin (GelMA) bioink. The use of GelMa as bioink is quite widespread because of its marked biocompatibility and biodegradability before and after 3D printing. This methodology demonstrated that the created environment improved the neural network formation and gene expression of neuronal biomarkers, such as Nestin or tubulin, compared to conventional 2D systems (visualized by immunofluorescence).^[^ ^151^ ^]^ Joung et al. reproduced a section of the spinal cord by bioprinting both neuronal and oligodendrocytes progenitor cells within microchannels, demonstrating that the deposition of bioinks in complex 3D structures improves the cell maturation process and the neurite elongation.^[^ ^152^ ^]^ In a study by Ning et al., Schwann cells were encapsulated in a composite bioink consisting of alginate, hyaluronic acid, and RGD and printed to evaluate nerve regeneration, demonstrating that 3D structure successfully drove neurite emission.^[^ ^153^ ^]^ In a recent work by De La Verga and colleagues, a fibrin‐based bioink loaded with microspheres was developed for the controlled release of molecules that are homogeneously distributed in the printed constructs.^[^ ^154^ ^]^ Following this approach, iPSCs were printed and differentiated within the printed constructs. The controlled release of the molecules and the reproduction of the 3D environment ensured successful differentiation of the cells into MNs. In Fantini's work, instead, a gelatin and sodium alginate‐based bioink was used to encapsulate three different cell types: SH‐SY5Y, iPSCs, and NSCs. All the three printed cell components manifested good viability up to 7 days and the scaffolds retained the printed shape over time.^[^ ^155^ ^]^ Ajiteru and colleagues developed an electroconductive bioink based on a reduced graphene oxide hydrogel combined with silk fibroin glycidyl methacrylate.^[^ ^156^ ^]^ This bioink showed adequate mechanical and neurogenic properties supporting the proliferation and vitality of Neuro2a, mouse neuroblast cells. Chen et al. developed a modular bioink consisting of GelMA/chitosan microspheres which was loaded with Schwann cells (RSC96) and PC12 cells and then printed.^[^ ^157^ ^]^ An environment suitable for the proliferation of Schwann cells and the creation of a neuronal network, as a potential model for the regeneration of nerve tissues, was obtained.

**Table 2:** Overview of works on the use of 3D bioprinter technology and hydrogels as bio‐inks for the study of NDDs.

Bioink	Cellular Type	Outcome	Reference
GelMA+dopamine	NSCs	Neuronal network formation and expression of neuronal markers	Zhou et al.^[^ ^151^ ^]^
Matrigel	Neuronal and oligodendrocyte progenitor	Cell maturation and neurite elongation	Joung et al.^[^ ^152^ ^]^
Alginate, hyaluronic acid, and RGD	Primary rat Schwann cells	Neurite elongation	Ning et al.^[^ ^153^ ^]^
Fibrin	iPSCs	Differentiation in motor neuronal cell	De la Vega et al.^[^ ^154^ ^]^
Gelatin and sodium alginate	iPSCs and NSCs, and SH‐SY5Y	High viability	Fantini et al.^[^ ^155^ ^]^
Silk fibroin glycidyl methacrylate	Neuro2a	Proliferation and viability	Ajiteru et al.^[^ ^156^ ^]^
GelMA/chitosan	Rat Schwann cells and PC12	Proliferation and formation of the neuronal network	Chen et al.^[^ ^157^ ^]^

## 4 3D Microfluidic In Vitro Systems

As mentioned before, the combination of microfluidic devices and 3D approaches represents an attractive prospect for modeling NDDs. Microfluidics offers the possibility to control spatially and temporally the microenvironment, while the presence of a 3D environment provides better cell survival, growth, and differentiation, allowing also long‐term experiments. By fusing together these two approaches, it is possible to overcome the drawbacks inherent each technique, achieving better results.^[^ ^158^ ^]^ There are three main possibilities:^[^ ^74^, ^75^, ^76^, ^77^, ^78^, ^79^, ^80^, ^81^, ^82^, ^83^, ^84^, ^85^, ^86^, ^87^, ^88^, ^89^, ^90^, ^91^, ^92^, ^93^, ^94^, ^95^, ^96^, ^97^, ^98^, ^99^, ^100^, ^101^, ^102^, ^103^, ^104^, ^105^, ^106^, ^107^, ^108^, ^109^, ^110^, ^111^, ^112^, ^113^, ^114^, ^115^, ^116^, ^117^, ^118^, ^119^, ^120^, ^121^, ^122^, ^123^, ^124^, ^125^, ^126^, ^127^, ^128^, ^129^, ^130^, ^131^, ^132^, ^133^, ^134^, ^135^, ^136^, ^137^, ^138^, ^139^, ^140^, ^141^, ^142^, ^143^, ^144^, ^145^, ^146^, ^147^, ^148^, ^149^, ^150^, ^151^, ^152^, ^153^, ^154^, ^155^, ^156^, ^157^, ^158^, ^159^ ^]^ i) manufacturing the mold through 3D printing, casting PDMS on 3D templates; ii) printing hydrogel‐embedded cells in a pre‐fabricated device; and iii) printing the entire chip device, including both cells and templates (i.e., microfluidic channels).

### 4.1 Manufacturing the Mold through 3D Printing, and Casting PDMS on 3D Templates

In this contest, 3D printers can be used to make molds and functional components for manufacturing classical PDMS devices.^[^ ^160^ ^]^ The production of unibody microfluidic platforms reduces the time and the cost necessary for conventional microfluidic chip processing that, in addition, requires a clean experimental environment and expensive equipment. Furthermore, this approach allows users to validate the design and the chip development cycle. Finally, with this method it is possible to maintain the excellent properties of PDMS, overcoming the troubles due to unknown surface properties of commercial resins.

### 4.2 Printing Hydrogel‐Embedded Cells in Pre‐Fabricated Device

Although the cell seeding process inside a microfluidic device is usually performed by hand or using a syringe pump, this approach is generally slow and can require an intricate setup made of pumps, tubing, and fluidic connections. Therefore, direct cell printing and/or patterning using a bioprinter would guarantee high‐throughput and high‐precision results, since the deposition of embedded cells in complicated 3D structures would allow the creation of personalized cell culture models to assess cell–cell and cell–matrix interactions. However, numerous challenges are still present. The limited resolution and high surface roughness of the most popular bioprinting strategy, that is, extrusion‐based printing, make bioprinting technology not appropriate for a microfluidic system with complicated heterogeneous 3D features. On the other hand, laser‐based SLA technology can reach high‐resolution, but suitable biomaterials are still in a limited number, and cells cannot be bioprinted simultaneously with the scaffold avoiding cell damage.

### 4.3 Printing the Entire Chip Device, Including Both Cells and Templates

This manufacturing method, often described as one‐step manufacturing, involves the building of the complete organ‐on‐chip by 3D printing, including the devices and the biological component. This means that complex 3D printing systems, capable of printing several materials (biomaterials and non‐biomaterials) simultaneously, must be developed. Although, this approach will allow the development of more relevant in vitro models, to date, many challenges need to be solved:^[^ ^74^, ^161^ ^]^ i) the precision and transparency of 3D‐printed microfluidic systems are generally not acceptable; and ii) the high accuracy 3D printing approaches are often not suitable for cell‐laden inks.

### 4.4 3D Microfluidic Platforms for Neurodegenerative Diseases

**Figure 6:**
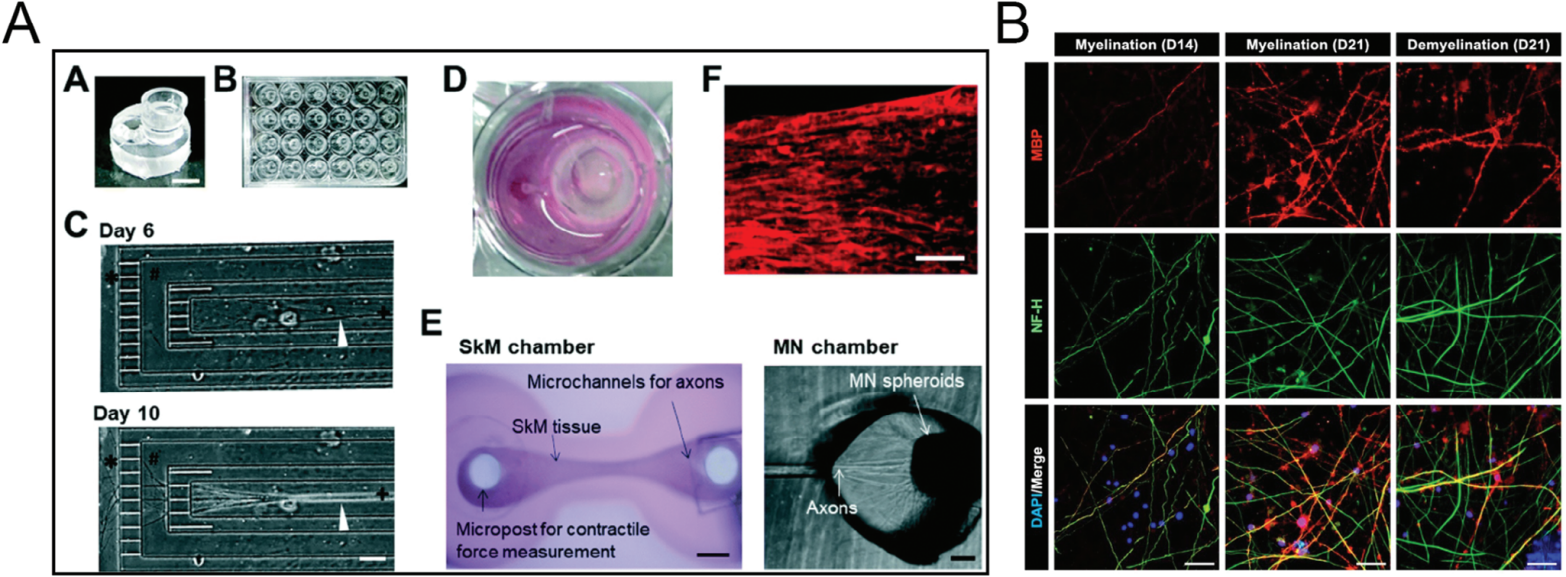
3D microfluidic in vitro systems for the study of NDDs. A) A human NMJ model in a 24‐well‐plate setup: A,B,D) axons from hiPSC‐derived MN spheroids extend in the MN chamber (C), reaching the muscular compartment (E,F). The force of engineered muscle tissues, whose contraction was triggered by MNs through the NMJ, was measured. Reproduced with permission,^[^ ^78d^ ^]^ Copyright 2021, Royal Society of Chemistry. B) A 3D PNS‐on‐chip to model myelin formation using ascorbic acid and demyelination by treating myelinated nerve fibers with glial growth factor (neuregulin‐1 isoform). Reproduced under the terms of the CC BY license,^[^ ^166^ ^]^ Copyright 2021, Springer Nature.

Neurons, glial cells but also muscle cells can be, at the same time, compartmentalized and embedded in specific hydrogels reproducing the complexity of the in vivo architectures. The concept of 3D devices can be controversial. The term “3D” can refer to different aspects: i) the manufacturing techniques of the device; ii) the presence of the ECM in which the cells are embedded; and iii) the ability to print the cells within the device. To date, in the neuroscience field, there are very few works in which “3D” was employed to refer to the technique used to produce the device. Johnson et al. fabricated a 3D printed nervous system‐on‐a‐chip using the micro‐extrusion 3D printing technique for the analysis of viral infection in the nervous system.^[^ ^162^ ^]^ 3D printing was used to pattern the surface introducing microchannels for favoring axonal alignment and creating confined chambers for cell separation. However, when referring to 3D microfluidic devices, devices in which cells are encapsulated in hydrogels that reproduce the ECM are often considered. Recently, many efforts have been devoted to the reproduction of the NMJ, damaged in NDDs such as ALS (**Figure** **6**A,B). One of the first 3D microfluidic NMJ model was proposed by Uzel et al.^[^ ^163^ ^]^ Combining microfluidics and optogenetic technology, they developed a physiologically relevant in vitro model of NMJ. Myoblast‐derived muscle strips and MNs derived from mouse embryonic stem cells were cocultured within a collagen–matrigel hydrogel and the presence of deformable pillars inside the muscle compartment allowed the quantification of the force generated by muscle contraction. Using a similar approach, Osaki et al., designed an ALS‐on‐a‐chip with iPSC‐derived MNs from a patient affected by sporadic ALS along with iPSC‐derived 3D muscle fiber constructs, embedded into a collagen–matrigel hydrogel.^[^ ^78a^ ^]^ ALS MNs were treated with bosutinib and rapamycin, drugs known to elicit a neuroprotection activity. In both the cases, neuronal survival and an intensified muscle contraction force were observed. Yamamoto et al. developed a microdevice able to fit in 24 well plates, presenting two chambers to host MNs and skeletal muscle (SkM) fibers, separately.^[^ ^78d^ ^]^ Both these cell types were embedded in a fibrin–matrigel hydrogel. The two chambers were connected by microtunnels, allowing axon elongation while preventing cell body migration. They evaluated muscle contraction after the supplementation of a neurotransmitter, glutamate, in the MN chamber. Moreover, they demonstrated how it is possible to probe not only the cell bodies of MNs and SkM cells, but also specific portions of the axons with chemical/mechanical stimuli (Figure 6C). The studies reported above investigated the interaction between MNs and muscle cells. However, in the in vivo motor unit there is a third critical cellular component represented by the terminal Schwann cells (TSCs) associated with the synapse.^[^ ^164^ ^]^ So far, protocols to generate pure population of TSCs still need to be developed. Therefore, even if various platforms have been proposed to reproduce NMJ, further improvements are needed to make the required cell models available. However, different groups investigated the interaction between rodent motor neurons and Schwann cells in 3D microfluidic platform generating models of PNS. Hyung et al. developed a 3D PNS microfluidic platform that resembles the myelination, demyelination, and remyelination phenomena.^[^ ^165^ ^]^ Using lysophosphatidylcholine, a molecule that is connected to dedifferentiation of readily differentiated SCs, they modeled the demyelination in vitro. In a second moment, they restored the myelin formation by using two drugs: benzatropine or methylcobalamin (Figure 6D). Park et al. proposed a micro‐engineered platform for modeling PNS myelination based on a MN–SC coculture in a collagen I hydrogel.^[^ ^166^ ^]^ By introducing ascorbic acid into the coculture model, they modeled the myelination process, resulting in higher expression levels of myelin markers in SCs. They also demonstrated that the process could be inverted by treating myelinated nerve fibers with neuregulin‐1 isoform, that is, a glial growth factor, resulting in demyelination.

3D microfluidic systems have also been employed to study other NDDs. The first 3D microfluidic in vitro brain model for NDDs dates back to 2015, when Park et al. developed a 3D AD model on a chip based on 3D neurospheroids cultured under a constant flow of fluid.^[^ ^167^ ^]^ They tested the neurotoxic effects of A*β* on neurospheroids in static and dynamic conditions. Results showed that A*β* treatment under continuous flow caused a decrease in neurospheroids viability, resulting in a significant destruction of neural network, if compared to treatment in static condition. Park et al. realized a 3D triculture system (neurons, astrocytes, and microglia) to reproduce neurodegeneration and inflammation in AD.^[^ ^78e^ ^]^ With this model, they evaluated the main hallmarks of the disease: A*β* aggregation, phosphorylated tau accumulation, and neuroinflammatory processes. Culturing AD neurons, they observed A*β* aggregates around the neurons and microglia migration toward the aggregates, inducing neuronal cell death around them (Figure 6E). Since the regrowth and the reconnection of the damaged neurons inside the nervous system is the final aim in curing NDDs, Tang et al. developed a 3D microfluidic device to study the regeneration of injured dopaminergic (DA) neurons in the presence of a chemical gradient.^[^ ^168^ ^]^ They cultured primary rat neurons in a 3D collagen hydrogel and then induced their degeneration using oxidopamine, a neurotoxic compound used to destroy dopaminergic and noradrenergic neurons. They observed that a gradient of three iridoid glycoside natural products promoted the axonal growth of DA neurons, also allowing an orientated repolarization and regeneration. Recent evidences have highlighted how neurovascular dysfunctions are linked to neuroinflammation and NDDs,^[^ ^169^ ^]^ therefore more relevant models of blood–brain barrier, able to imitate the complexity of the naïve function, need to be developed.^[^ ^170^ ^]^ Osaki et al. engineered a 3D vascular and neuronal circuit in a microfluidic device in order to study possible interactions between these two components.^[^ ^171^ ^]^ Using embryonic stem cells‐derived MNs and iPSCs‐derived endothelial cells (ECs) embedded in a collagen‐based hydrogel, they demonstrated that vascular network promotes neurite elongation and neural activity probably due to both paracrine signaling from ECs mediated by BDNF and direct juxtacrine signaling via the delta‐notch pathway.

## 5 Challenges and Future Perspectives

Although significant efforts have been dedicated in these years to the understanding of the pathological mechanisms of NDDs, these remain largely unknown due to the complexity of the nervous system and the difficulty of obtaining cells directly from the patients (i.e., neurons, glial cells). Therefore, the reconstitution of in vivo like physiology and pathology in vitro continue to represent the major obstacle for the study of these diseases, resulting also in the discovery of new drugs and the development of therapeutic approaches. The growing interest in microfluidics and the possibility to combine this technology with a variety of ECM biomaterials could be the next frontier to obtain in vitro models more similar to the in vivo conditions. Although OoCs have several advantages, some technical and economic limitations must be still tackled. For example, most of the publications focused on the development of static devices rather than dynamic ones: this is largely related to the difficulties in introducing a fluid flow stress in neuronal culture. Cells in the brain are particularly sensitive to shear stress phenomena and even a small movement in the seeding or maintenance could lead to cell detachment. Minimizing this effect could be achieved by combining the spheroid/organoid culture with the microfluidic one. Organoids show indeed higher resistance to shear stress, and fluid flow can therefore be integrated even in the very early stage of differentiation. On the other hand, this could be useful to overcome the perfusion problems faced by conventional organoid cultures.^[^ ^28b^ ^]^ Finally, the integration of patient organoid‐derived cells inside microfluidic platforms could lead to powerful in vitro preclinical models in the future.

Future efforts will likely focus on making these devices more standardized, to be reliably fabricated on an industrial scale, and more than anything increasing their user‐friendly capability in order to be largely employed also by non‐specialist end users. The need to produce OoCs on a large‐scale calls also for the development of new manufacturing techniques, to increase the production speed, and new biocompatible materials, to fabricate the final platforms. In this regard, additive manufacturing technologies such as 3D printing are increasingly being implemented due to their advantages: i) the possibility to speed up the production process; ii) the capacity to rapidly prototype new OoC layouts having a profound impact on how researchers develop their platforms, as an iterative improvement can be achieved promptly and successfully. Another important obstacle in the acceptance of OoCs in the academic world at large is represented by the amount and quality of biological data that can be obtained from each experiment. However, growing concerns about using animals as ideal models for scientific research and their low predictivity led to a history‐making decision in America, recently. In late December 2022, President Joe Biden signed a legislation according to which new drugs need not be tested in animal models to obtain US Food and Drug Administration approval.^[^ ^172^ ^]^ This decision could provide a further incentive in the development and use of OoCs as valid methods for studying and testing new drugs.

The ideal in vitro model for NDDs should combine all the advantages of these different technologies: the cell self‐organization present in organoids, the accurate control over cell seeding, the cell compartmentalization, and the ability to recreate mechanical forces in microfluidic devices. We are probably still far from this goal and many other efforts will have to be made, but those made so far prove that researchers are going in the right direction.

## Conflict of Interest

The authors declare no conflict of interest.

## Author Contributions

Conceptualization: A.P. and F.G. Writing the original draft: E.D.V and A.S. Reviewing and editing the original draft: F.G., L.M., and A.P. Funding acquisition: A.P. and G.G. All authors reviewed the manuscript.
